# Effects of Surface Stability on Muscle Activation during Plank Exercise

**DOI:** 10.5114/jhk/203417

**Published:** 2025-09-23

**Authors:** Ali F. Sağlam, Erbil M. Aydın, Erkan Demirkan, Burak Gündoğan, Michael Favre

**Affiliations:** 1 Institute of Postgraduate Studies, Çanakkale Onsekiz Mart University, Çanakkale, Turkey.; 2 Faculty of Sport Sciences, Hitit University, Çorum, Turkey.; 3 Intercollegiate Athletics, University of Michigan, Ann Arbor, Michigan, USA.

**Keywords:** electromyography, stable surface, unstable surface, rectus abdominis, anterior deltoid

## Abstract

This study aimed to compare rectus abdominis (RA) and anterior deltoid (AD) muscle activation during plank exercise performed on different unstable surfaces. Sixteen recreationally active males participated in this study. A ground surface was used as a stable surface (SS), whereas gymnastics rings (GR) and the Bosu ball (BB) were used as unstable surfaces. Under the dual instability (DI) condition, participants placed their feet in the GR and elbows on the BB. Participants performed plank exercise under four different conditions. Muscle activation was measured in the upper rectus abdominis (URA), lower rectus abdominis (LRA), and anterior deltoid (AD) muscles during plank exercise. One-way repeated-measures analysis of variance was used to statistically compare muscle activation among the conditions. The level of significance was set at p < 0.05. Analysis showed that URA muscle activity was significantly greater under the DI condition than under the SS and BB conditions. In addition, URA muscle activity was greater under the GR than the SS condition. LRA muscle activity was significantly greater under the DI condition compared to the other conditions. In contrast, AD muscle activity was greater under the SS than the DI condition. In conclusion, these findings indicated that RA muscle activation was greatest on the dual instability surface, while AD muscle activation was greatest on the stable surface. Dual instability plank exercise may be a better choice than single instability and stable surface plank exercise for URA and LRA activation.

## Introduction

The core region, which includes the transversus abdominis, multifidus, and internal and external oblique muscles, contributes to trunk stabilization (Kim et al., 2016). It is important for maintaining spinal posture and for proper control of movements in functional activities. In addition, it has an important place in the prevention and treatment of lumbar injuries, including low back pain (Babakhani and Hatefi, 2019). The core region plays an important role in providing force transfer between the lower and the upper body. Achieving postural control requires stabilization and strength of the core region. Postural control is important for transferring energy from one segment of the body to another during movement. Lack of postural control may cause problems in energy transfer to the distal segments (Oliver et al., 2010). Therefore, this region needs to be strengthened to improve sports performance. Additionally, core exercises contribute to improved athletic performance and might reduce the risk of injury (Loturco et al., 2024). Strengthening the trunk or core stabilization muscles is important not only for athletic performance, but also for activities of daily living and rehabilitation of low back pain (Behm and Anderson, 2006). Several dynamic and static exercises can be performed to strengthen this region.

The inclination towards exercises aimed at strengthening or stabilizing the core muscles has been increasing, with many fitness and exercise programs including them (Mirmohammad et al., 2019; Schoenfeld and Contreras, 2013; Yoo, 2016). However, exercises involving spinal flexion, such as curl-ups, are claimed to pose a risk of injury to the lumbar region owing to excessive loading. Consequently, isometric exercises such as planks have become preferred (Peterson, 2013).

Plank exercise, which is performed using body weight and imposes minimal stress on the back, is an important type of exercise designed to enhance endurance, strength, and stability of the core muscles (Snarr et al., 2016). In recent years, there has been a growing recognition of the importance of plank exercises, leading to experiments with different variations to increase the activation level of the core muscles. One of the most preferred techniques is plank exercise using instability devices (Babakhani and Hatefi, 2019; Byrne et al., 2014; Mirmohammad et al., 2019; Topçu et al., 2022). These devices aim to increase the effort of the core muscles to maintain balance and spinal stability by creating instability during exercise, resulting in higher muscle activation than traditional exercises. Thus, they are used to increase the workload on the muscles involved in maintaining the body's stability and balance (Snarr et al., 2016).

Topçu et al. (2022) compared activation of core muscles during plank exercise using instability devices (such as a Bosu ball, a Swiss ball, and a TRX Suspension Trainer) with that performed on a stable surface without such devices. They found that plank exercise on the TRX Suspension Trainer and a Swiss ball resulted in higher activation than that on a stable surface and a Bosu ball. Moreover, Babakhani and Hatefi (2019) observed significantly higher muscle activation during side plank exercise on an unstable surface, particularly on a Swiss ball, than during exercises on a stable surface. Similarly, Snarr and Esco (2014) noted that higher muscle activation was obtained during plank exercise using instability devices such as a Swiss ball and a TRX Suspension Trainer compared to this exercise performed on a stable surface.

Based on previous studies, it can be anticipated that increasing surface instability leads to higher muscle activation. TRX-suspended apparatus has been widely used to achieve unstable surfaces. The ring apparatus is a suspended apparatus with a wide range of motion. Therefore, it can be used to obtain an unstable surface during exercise. This study aimed to compare activation levels of the anterior deltoid and rectus abdominis muscles during plank exercise performed under four different surface conditions. We hypothesized that the greatest anterior deltoid and rectus abdominis muscle activation would be achieved under the mixed condition, in which the elbows were placed on the Bosu ball and the feet were placed in the ring apparatus.

## Methods

### Participants

Sixteen males (age: 19.81 ± 1.56 years; body height: 176.41 ± 6.64 cm; body mass: 72.83 ± 8.24 kg; body fat percentage: 13.40 ± 4.82%) participated in this study. All participants signed an informed consent form before the start of the study. This study was approved by the ethics committee of the Hitit University, Çorum, Turkey (approval number: 2022-28; approval date: 09 January 2023). The study was conducted according to the Declaration of Helsinki.

### Measures

**Figure 1:**
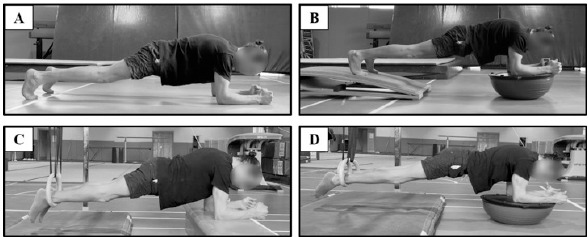
Plank exercises. A: Stable, B: Bosu Ball, C: Gymnastics rings, D: Dual instability.

Participants performed plank exercise on four different surfaces, including the feet and forearms on a stable surface (SS) (Figure 1A), feet on the stable surface, while forearms on a Bosu ball (BB) (Figure 1B), feet in the gymnastics rings (GR), while forearms on the SS (Figure 1C), and feet in the GR, while forearms on the BB [Dual Instability (DI)] (Figure 1D). Description of the plank exercise variations is provided in Table 1. Plank exercise lasted 10 s for each condition. Participants performed two sets of plank exercise on each surface. Three-minute rest intervals were provided between sets and conditions.

A Delsys Trigno 4-channel EMG device (Delsys Inc., Boston, MA, USA) was used for muscle activity measurements. The electrodes were placed on the upper rectus abdominis (URA), lower rectus abdominis (LRA), and anterior deltoid (AD) muscles. The electrode was placed approximately 2 cm laterally and across from the umbilicus parallel to the muscle fibers over the muscle belly for the URA (Brown et al., 2024; Calatayud et al., 2017a). The other electrode was placed parallel to the muscle fibers and near the midpoint between the umbilicus and the pubic symphysis and 3 cm laterally from the midline over the muscle belly for the LRA (Calatayud et al., 2017a). The electrode was placed at one finger width distal and anterior to the acromion for the AD (Campos et al., 2020). The EMG signals were sampled at approximately 1926 Hz and bandpass filtered at 20–450 Hz. Two trials of maximum isometric voluntary contraction (MVIC) measurements were performed before plank exercises. Each MVIC trial lasted 5 s. In the normalization calculation, the average of the root mean square (RMS) values of the middle three seconds was used by subtracting 1 s from the beginning and the end of the two MVIC trials (Slater and Hart, 2017). MVIC tests were performed as follows: for URA and LRA muscles, participants were placed in a supine position on the ground with knees and hips flexed at 90° and feet supported. Participants were asked to maximally flex their trunks (as when performing a crunch) while the researcher provided manual resistance to the participants' shoulders (Escamilla et al., 2010). For the anterior deltoid, participants were asked to flex their shoulders 90° while sitting without back support. In this position, participants were asked to exert an upward force against a fixed bar (Calatayud et al., 2014). Verbal encouragement was provided to all participants during the MVIC tests.

The root mean square (RMS) values were used in the analysis. In the RMS analysis, the first and the last second of the raw EMG data obtained from the plank exercise were discarded and the remaining 8 s were considered. The peak RMS values of the two plank trials were used for normalization. Peak RMS values were normalized to the RMS values obtained from MVIC. Therefore, all data are presented in % MVIC.

### Design and Procedures

A crossover experimental design model was used to compare the four conditions in this study. Trials of plank exercise under four different conditions were performed before starting the study. A one-week rest period was given to participants after the familiarization session. The ground was used as a stable surface and the GR, the BB, and a mixture of the GR and the BB (DI) were used as an unstable surface.

### Statistical Analysis

The mean and standard deviation (X ± SD) values of the data were used in this study. The normal distribution characteristics of the data were examined using the Shapiro-Wilk normality test. One-way repeated-measures analysis of variance was applied for statistical analysis of the data. A significance level of *p* < 0.05 was accepted for all statistical analyses. All analyses were performed using the SPSS 25 program.

## Results

Table 2 presents the activities of the URA, LRA, and AD muscles on different surfaces. Significant differences were found for all muscles activity among the four different conditions. The highest muscle activity for the URA and LRA was observed under the DI condition. The URA muscle activity under the DI condition was greater than under the BB (*p* = 0.007) and SS (*p* = 0.003) conditions. No significant difference was found between the DI and GR conditions. In addition, URA muscle activity under the GR condition was greater than under the SS condition (*p* = 0.045). LRA muscle activity was greater under the DI condition compared to the GR (*p* = 0.018), BB (*p* = 0.002), and SS (*p* = 0.001) conditions. AD muscle activity under the SS condition was greater than under the DI condition (*p* = 0.005).

**Table 1:** Description of plank variations performed on different surfaces.

Plank Exercise Variation	Description
Plank on a stable surface (Figure 1A)	Participants were asked to maintain their position with their head in a neutral position and their elbows directly beneath their glenohumeral joint. Participants kept their elbows and feet shoulder-width apart. Participant kept the body as straight as possible for 10 s.
Plank with the feet on a stable surface and forearms on the Bosu ball (Figure 1B)	Participants were asked to maintain their position with their head in a neutral position and their elbows directly beneath their glenohumeral joint. They kept their elbows and feet shoulder-width apart. For each participant, the foot and elbow heights were adjusted using a springboard to provide the same body inclination as on the stable surface. Participants kept the body as straight as possible for 10 s.
Plank with the feet in the gymnastics rings and forearms on a stable surface (Figure 1C)	Participants were asked to maintain their position with their head in a neutral position and their elbows directly beneath their glenohumeral joint. They kept their elbows and feet shoulder-width apart. The handles of the gymnastic rings were adjusted to be 35 cm above the floor. Thus, the foot and elbow heights were adjusted to provide the same body inclination as on the stable surface. Participants kept the body as straight as possible for 10 s.
Plank with the feet in the gymnastics rings and forearms on the Bosu ball (Dual Instability) (Figure 1D)	Participants were asked to maintain their position with their head in a neutral position and their elbows directly beneath their glenohumeral joint. They kept their elbows and feet shoulder-width apart. The handles of the gymnastic rings were adjusted to be 35 cm above the floor. Thus, the foot and elbow heights were adjusted to provide the same body inclination as on the stable surface. Participants kept the body as straight as possible for 10 s.

**Table 2:** Normalized (% MVIC) muscle activation obtained during plank exercise performed on different surfaces.

	SS	BB	Δ%	GR	Δ%	DI	Δ%
**URA**	36.23 ± 19.85*^#^	39.86 ± 22.26*	10.02	57.89 ± 38.45	59.78	77.23 ± 48.97	113.17
**LRA**	35.3 ± 17.78*	37.11 ± 17.25*	5.13	48.57 ± 27.11*	37.59	63.85 ± 30.9	80.88
**AD**	29.63 ± 12.47*	27.55 ± 14.73	−7.02	24.16 ± 9.49	−18.46	19.37 ± 6.55	−34.63

SS: Stable Surface, BB: Bosu Ball, GR: Gymnastics Rings, DI: Dual Instability, URA: Upper Rectus Abdominis, LRA: Lower Rectus Abdominis, AD: Anterior Deltoid. Δ%: Percent difference according to a stable surface. * significant difference according to DI; ^#^ significant difference according to GR

**Figure 2:**
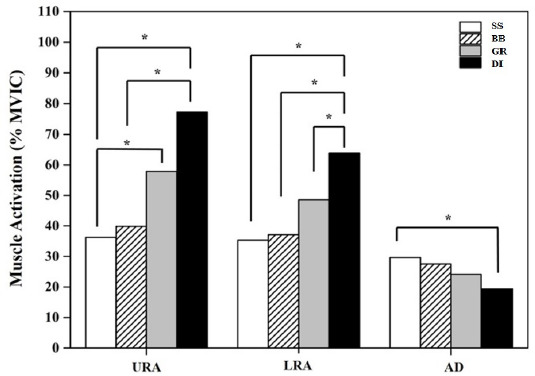
Comparison of normalized muscle activity of URA, LRA, and AD muscles among the four conditions. * p < 0.05

## Discussion

In resistance exercises, the level of motor unit activity stimulated during movement execution is one of the most basic criteria for evaluating the efficiency of the target muscle group. In other words, the greater the motor unit stimulation occurring during the execution of a movement in resistance exercise, the higher the expected efficiency of training. In recent years, many studies have been conducted with the focus on the classically applied resistance exercises in their different variations. Those studies made significant contribution to the literature in terms of evaluating exercise efficiency. Therefore, this study aimed to compare the electromyographical (EMG) muscle activation of the rectus abdominis (RA) upper/lower and anterior deltoid muscle fibril, obtained during plank exercise performed on surfaces with different levels of stability. The most important finding of this study was that the greatest EMG output occurred in the upper (113.17%) and the lower (80.88%) RA while performing the plank on unstable surfaces using the Bosu ball and ring devices (dual) compared with the traditional method (floor plank) (Table 2, Figure 2). To the best of our knowledge, this is the first study to utilize dual variations with rings and a Bosu ball. In previous studies, TRX and suspension devices have been commonly used for instable plank variations. Based on these results, the gymnastics rings may also be considered an effective training apparatus for the core area. Besides, our results showed that the other plank variations using devices such as a Bosu ball and gymnastics rings provided more muscle activation in the upper (10.02% and 59.78%, respectively) and the lower (5.13% and 37.59%, respectively) RA than the traditional plank exercise performed on a stable surface (Figure 2). The results of this study are consistent with previous research regarding instability variations of the plank while using devices, and reveal higher EMG activity of the RA compared with traditional plank exercises designed to target the abdominal wall (Cugliari and Boccia, 2017; Park and Park, 2019; Snarr and Esco, 2014; Topçu et al., 2022). For instance, Snarr and Esco (2014) demonstrated increased rectus abdominis (RA), external oblique (EO), and lumbosacral erector spinae muscle activation when planks were performed on a Swiss ball and TRX compared with the traditional plank. Byrne et al. (2014) reported that suspension training using instability devices (TRX Suspension System) in plank variations, significantly increased the magnitude of muscle activation in the RA and EO muscles. Topçu et al. (2022) stated that the activity of the RA and EO muscles during plank exercise was higher when using the TRX than when using a Swiss ball, a Bosu ball, and a stable surface. In addition, those authors concluded that using devices such as the TRX, the Swiss ball, and the Bosu ball was more effective in core muscle activation during plank exercise than a stable surface. In another study of different plank variations without instability devices conducted by Schoenfeld et al. (2014), the long-lever posterior-tilt plank movement resulted in more muscle activation in the upper-lower RA and EO, but not in the erector spinae muscle, when compared to the traditional prone plank. In a similar study, Lee et al. (2017) investigated trunk muscle activation with postural changes during plank exercise using a dynamic cushion. Those authors found that an unstable traditional plank exercise produced relatively greater muscle activation than the other variations. Babakhani and Hatefi (2019) reported that side plank exercise using Swiss balls as an unstable surface led to significant changes in the EMG activity of the gluteus medius, RA, and EO muscles when compared to stable surfaces. Park and Park (2019) compared traditional plank exercise and bilateral leg raise movements, and their results indicated that the rectus abdominis had greater activation (45% maximal voluntary contraction) during the bilateral leg raise exercise when compared to the plank exercise. Snarr et al. (2016), while investigating the effects of prone pike movement variations on trunk muscles, reported that using instability devices was more effective at enhancing EMG activation in the RA, EO, rectus femoris, and erector spinae muscles when compared to a stable prone pike. Lehman et al. (2005) stated that higher EMG activity occurred in the RA and EO muscles during the plank with the Swiss ball than during traditional plank exercise. Cugliari and Boccia (2017) demonstrated greater activation of RA muscle in the roll-out exercise, which is a type of a plank performed using a suspension device, than in the other variations. In contrast to these findings, Mirmohammad et al. (2019) conducted a study that examined differences in sonographic activity of the deep lumbopelvic muscles during plank exercise in female athletes. Those authors reported no changes in the level of deep core muscle activity during front and side plank exercise on stable and unstable surfaces. According to all these studies, except for the aforementioned Mirmohammad et al. (2019), the results suggest that using unstable surfaces and devices results in higher EMG activity of the core muscles in the abdominal wall compared to a traditional stable plank. The literature and our study findings demonstrate that instability devices such as suspension devices (including a TRX Suspension Trainer and gymnastics rings), a Swiss ball, and a Bosu ball should be used to obtain more efficiency in strengthening the abdominal wall muscles compared to the traditional plank performed on a stable surface.

Another interesting result of this study was that the EMG output of the anterior deltoid was higher on a stable surface compared to the DI condition (Table 2, Figure 2). This decrease in EMG output in the anterior deltoid (AD) during unstable exercises could be due to forward and backward movements with the elbows while on unstable surfaces during the plank position, or not being a prime mover muscle for plank exercise because of the distance of the center of the core area. In a study related to supine plank positions, Calatayud et al. (2017b) investigated trunk muscle EMG activity during supine plank exercise in suspended and stable bilateral and unilateral positions. Those authors found no differences between exercises for the upper/lower rectus abdominis and EO muscle activity for all exercise variations, except for lumbar erector spinae muscle activity (Calatayud et al., 2017b). A similar study conducted by Lehman et al. (2005) compared supine and prone plank EMG activity, and their results showed that EMG activity in RA muscle was higher in the prone plank position than in a supine plank (26.6 vs. 5.84). The reason for this finding is that the upper-lower RA and EO muscles may not be the prime movers for the supine plank exercise.

## Conclusions

Our study results indicate that greater voluntary muscle contraction occurs in the upper and lower RA motor unit activation when the plank is performed under a dual-instability condition using the gymnastics rings and a Bosu ball compared to the traditional plank performed on a stable surface and its other variations. Based on these findings, dual plank exercise may be a better choice for upper and lower RA activation. It should be noted that athletes with weak abdominal strength and endurance may benefit from the dual plank using a Bosu ball and gymnastics rings, which could be considered a core training alternative. However, dual instability plank variation leads to lower EMG activation compared to the traditional stable plank for the anterior deltoid.

## References

[ref1] Babakhani, F., & Hatefi, M. (2019). Comparing the electromyography activity of core muscles during side plank exercise on stable and unstable surfaces. *Journal of Sport Biomechanics*, 5(2), 102–111. 10.32598/biomechanics.5.2.3

[ref2] Behm, D. G., & Anderson, K. G. (2006). The role of instability with resistance training. *Journal of Strength & Conditioning Research*, 20(3), 716–722. 16937988 10.1519/R-18475.1

[ref3] Brown, M. B., Peters, R., & Lauder, M. A. (2024). Contribution of Trunk Rotation and Abdominal Muscles to Sprint Kayak Performance. *Journal of Human Kinetics*, 90, 5–15. 10.5114/jhk/169939 38380295 PMC10875689

[ref4] Byrne, J. M., Bishop, N. S., Caines, A. M., Crane, K. A., Feaver, A. M., & Pearcey, G. E. (2014). Effect of using a suspension training system on muscle activation during the performance of a front plank exercise. *Journal of Strength & Conditioning Research*, 28(11), 3049–3055. 10.1519/JSC.0000000000000510 24796979

[ref5] Calatayud, J., Borreani, S., Colado, J. C., Martín, F. F., Rogers, M. E., Behm, D. G., & Andersen, L. L. (2014). Muscle activation during push-ups with different suspension training systems. *Journal of Sports Science & Medicine*, 13(3), 502–510. 25177174 PMC4126284

[ref6] Calatayud, J., Casaña, J., Martín, F., Jakobsen, M. D., Colado, J. C., & Andersen, L. L. (2017a). Progression of core stability exercises based on the extent of muscle activity. *American Journal of Physical Medicine & Rehabilitation*, 96(10), 694–699. 10.1097/PHM.0000000000000713 28157133

[ref7] Calatayud, J., Casaña, J., Martín, F., Jakobsen, M. D., Colado, J. C., Gargallo, P., & Andersen, L. L. (2017b). Trunk muscle activity during different variations of the supine plank exercise. *Musculoskeletal Science and Practice*, 28, 54–58. 10.1016/j.msksp.2017.01.011 28171779

[ref8] Campos, Y. A., Vianna, J. M., Guimarães, M. P., Oliveira, J. L., Hernández-Mosqueira, C., da Silva, S. F., & Marchetti, P. H. (2020). Different shoulder exercises affect the activation of deltoid portions in resistance-trained individuals. *Journal of Human Kinetics*, 75, 5–14. DOI: 10.2478/hukin-2020-0033 33312291 PMC7706677

[ref9] Cugliari, G., & Boccia, G. (2017). Core muscle activation in suspension training exercises. *Journal of Human Kinetics*, 56(1), 61–71. DOI: 10.1515/hukin-2017-0023 28469744 PMC5384053

[ref10] Escamilla, R. F., Lewis, C., Bell, D., Bramblet, G., Daffron, J., Lambert, S., & Andrews, J. R. (2010). Core muscle activation during Swiss ball and traditional abdominal exercises. *Journal of Orthopaedic & Sports Physical Therapy*, 40(5), 265–276. https://www.jospt.org/doi/10.2519/jospt.2010.3073 20436242 10.2519/jospt.2010.3073

[ref11] Kim, S. Y., Kang, M. H., Kim, E. R., Jung, I. G., Seo, E. Y., & Oh, J. S. (2016). Comparison of EMG activity on abdominal muscles during plank exercise with unilateral and bilateral additional isometric hip adduction. *Journal of Electromyography and Kinesiology*, 30, 9–14. 10.1016/j.jelekin.2016.05.003 27213781

[ref12] Lee, D., Lee, Y., Cho, H. Y., Lee, K. B., Hong, S., Pyo, S., & Lee, G. (2017). Investigation of trunk muscle activity for modified plank exercise: a preliminary study. *Isokinetics and Exercise Science*, 25(3), 209–213. 10.3233/IES-171113

[ref13] Lehman, G. J., Hoda, W., & Oliver, S. (2005). Trunk muscle activity during bridging exercises on and off a Swissball. *Chiropractic & Osteopathy*, 13, 1–8. 10.1186/1746-1340-13-14 16053529 PMC1187901

[ref14] Loturco, I., Zabaloy, S., Pereira, L. A., Moura, T. B. M. A., Mercer, V. P., Fernandes, V. ... & Bishop, C. (2024). Resistance Training Practices of Brazilian Olympic Sprint and Jump Coaches: Toward a Deeper Understanding of Their Choices and Insights (Part III). *Journal of Human Kinetics*, 90, 183–214. 10.5114/jhk/182888 38380293 PMC10875694

[ref15] Mirmohammad, R., Minoonejhad, H., & Sheikhhoseini, R. (2019). Ultrasonographic comparison of deep lumbopelvic muscles activity in plank movements on stable and unstable surface. *Physical Treatments-Specific Physical Therapy Journal*, 9(3), 147–152. 10.32598/ptj.9.3.147

[ref16] Oliver, G. D., Dwelly, P. M., Sarantis, N. D., Helmer, R. A., & Bonacci, J. A. (2010). Muscle activation of different core exercises. *Journal of Strength & Conditioning Research*, 24(11), 3069–3074. 10.1519/JSC.0b013e3181d321da 20733527

[ref17] Park, D. J., & Park, S. Y. (2019). Which trunk exercise most effectively activates abdominal muscles? A comparative study of plank and isometric bilateral leg raise exercises. *Journal of Back and Musculoskeletal Rehabilitation*, 32(5), 797–802. 10.3233/BMR-181122 30856100

[ref18] Peterson, D. D. (2013). Proposed performance standards for the plank for inclusion consideration into the navy's physical readiness Test. *Strength & Conditioning Journal*, 35(5), 22–26. 10.1519/SSC.0000000000000003

[ref19] Schoenfeld, B. J., & Contreras, B. M. (2013). The long-lever posterior-tilt plank. *Strength & Conditioning Journal*, 35(3), 98–99. 10.1519/SSC.0b013e31828226d5

[ref20] Schoenfeld, B. J., Contreras, B., Tiryaki-Sonmez, G., Willardson, J. M., & Fontana, F. (2014). An electromyographic comparison of a modified version of the plank with a long lever and posterior tilt versus the traditional plank exercise. *Sports Biomechanics*, 13(3), 296–306. 10.1080/14763141.2014.942355 25325773

[ref21] Slater, L. V., & Hart, J. M. (2017). Muscle activation patterns during different squat techniques. *Journal of Strength & Conditioning Research*, 31(3), 667–676. 10.1519/JSC.0000000000001323 26808843

[ref22] Snarr, R. L., & Esco, M. R. (2014). Electromyographical comparison of plank variations performed with and without instability devices. *Journal of Strength & Conditioning Research*, 28(11), 3298–3305. 10.1519/JSC.0000000000000521 25264667

[ref23] Snarr, R. L., Hallmark, A. V., Nickerson, B. S., & Esco, M. R. (2016). Electromyographical comparison of pike variations performed with and without instability devices. *Journal of Strength & Conditioning Research*, 30(12), 3436–3442. 10.1519/JSC.0000000000001436 27191692

[ref24] Topçu, H., Arabacı, R., Güngör, A. K., Birinci, Y. Z., Pancar, S., & Şekir, U. (2022). Muscle activity of core muscles during plank exercise on different surfaces. *Turkish Journal of Sport and Exercise*, 24(3), 298–305.

[ref25] Yoo, K. T. (2016). The effect of flexibility of bridge and plank exercises using sling suspension on an unstable surface on while standing in healthy young adults. *Journal of the Korean Society of Physical Medicine*, 11(3), 1–9. 10.13066/kspm.2016.11.3.1

